# Identification and Pathogenic Potential of *Bacillus cereus* Strains Isolated from a Dairy Processing Plant Producing PDO Taleggio Cheese

**DOI:** 10.3390/microorganisms8060949

**Published:** 2020-06-24

**Authors:** Erica Tirloni, Simone Stella, Cristian Bernardi, Diletta Mazzantini, Francesco Celandroni, Emilia Ghelardi

**Affiliations:** 1 Department of Health, Animal Science and Food Safety, University of Milan, via Celoria 10, 20133 Milan, Italy; erica.tirloni@unimi.it (E.T.); simone.stella@unimi.it (S.S.); cristian.bernardi@unimi.it (C.B.); 2 Department of Translational Research and New Technologies in Medicine and Surgery, University of Pisa, Via San Zeno 37, IT-56127 Pisa, Italy; diletta.mazzantini@med.unipi.it (D.M.); francesco.celandroni@dps.unipi.it (F.C.); 3 Research Center Nutraceuticals and Food for Health-Nutrafood, University of Pisa, 56128 Pisa, Italy

**Keywords:** *Bacillus cereus*, dairy plant, taleggio cheese, virulence, biofilm

## Abstract

Low levels of contamination by *Bacillus cereus* at the cheese farm is essential for reducing any opportunity for growth prior consumption. In this study, *B. cereus* distribution in a plant producing Protected Designation of Origin Taleggio cheese was investigated and the virulence potential of the isolates was evaluated. Seventy-four samples were collected from Food and Non Food Contact Surfaces (FCS, NFCS), saline curd, and Taleggio. The eleven isolates were identified, typified, and clustered. Strains were tested for the production of hemolysins, hemolysin BL (HBL), phosphatidylcholine-specific phospholipase C (PC-PLC), proteases, and biofilm, and for the presence of chromosomal toxin-encoding genes (*sph*, *plcA*, *cytK*, *entFM*, *bcet*, *entS*, *nheA*, *nheB,* *nheC*). *B. cereus* was detected on NFCS, FCS, and curd, but not in Taleggio. The isolates were grouped into six clusters, and all produced PC-PLC, hemolysins, and proteases, and most of them HBL (66.7%). All the clusters harbored the *nheA, sph, plcA*, *entFM*, and *cytK* genes, and some also *nheB* (83.3%)*,* *nheC* (66.7%), *bcet* (50.0%), and *entS* (66.7%). All strains showed biofilm-forming ability. Our data reveal possible contamination of production plants and cheese curd by potentially virulent *B. cereus*, but bacterial absence in Taleggio highlights the efficacy of a proper management of the production phases in assuring consumer’s protection.

## 1. Introduction

Spore forming bacteria are still a challenge in dairy processing plants in terms of dairy product safety and spoilage. In particular, *Bacillus cereus* is recognized as a common contaminant of dairy products with frequent isolation from raw and pasteurized milk [1,2,3], as well as from final products [4,5]. This microorganism was also proven able to replicate in some dairy products such as ricotta and mascarpone cheese [6,7].

*B. cereus* is recognized as an important spoilage microorganism [8], because of the production of extracellular enzymes like proteases, lipases, lecithinases, and phospholipases, which hydrolyze caseins and triacylglycerols or determine a disruption of the fat globule structure, leading to degradation of milk components and additives [9]. *B. cereus* is associated with two types of diseases, namely the emetic syndrome (determined by cereulide, a small ring-shaped peptide pre-formed in food before consumption) and the diarrheal syndrome (caused by one or more enterotoxins produced in the small intestine by live bacteria) [10,11,12].

In dairy plants, the ability of *B. cereus* to adhere to surfaces and form biofilms is of great concern for manufacturers, since biofilm can be a constant source of recurrent contamination of products during the production stages [9].

The aim of this study was to monitor the presence of *B. cereus* in a dairy plant producing Protected Designation of Origin (PDO) Taleggio cheese. Taleggio is a soft cheese produced in a specific area (8 provinces) of Northern Italy. It is made of whole raw or pasteurized cow milk. The curd is obtained by the addition of calf rennet and microbial starters (*Lactobacillus bulgaricus* and *Streptococcus thermophilus*) at a temperature of 32–35 °C. After breaking and purging, the curd is distributed into the molds; it is submitted to a resting phase at 22–25 °C that lasts for 8–16 h and to dry- or brine-salting. Then, ripening takes place on wood axes or boxes and lasts for a minimum of 35 days at refrigeration temperatures. During this phase, the cheese is weekly turned and sponged with salt water. Differently from other soft cheeses, the rind is intended for consumption. In order to monitor the diffusion of *B. cereus*, all the areas of the plant were sampled, considering several different points. All presumptive *B. cereus* isolates were identified by matrix assisted laser desorption/ionization-time of flight mass spectrometry (MALDI-TOF MS) and typified by randomly amplified polymorphic DNA PCR (RAPD-PCR), to better comprehend *B. cereus* spread among different sites of the dairy plant. Moreover, the putative pathogenic significance of *B. cereus* contamination was investigated by evaluating the production of hemolysins, phospholipases, proteases, and the ability to form biofilms, as well as the presence of toxin-encoding genes of the isolated bacterial clusters.

## 2. Materials and Methods

### 2.1. Experimental Plan

Environmental samples were collected from a medium-scale dairy industry producing PDO Taleggio cheese, during two sampling sessions performed in May and July 2017. The plant included two main sections, intended for cheese production and for ripening. A total of 74 environmental samples were collected during production from different areas (production, brining/ripening, packaging). The sampling points were identified as food contact surfaces (FCS), transfer-non food contact surfaces (tr-NFCS, points that can favor the transfer of the contamination among different areas), and non-transfer-NFCS (non-tr-NFCS). Several points were sampled, as summarized in Table 1. In addition to environmental samples, three samples of salt/saline, two samples of curd, and four samples of Taleggio rinds (at the end of the ripening period) were taken.

### 2.2. Sample Analysis

Sterile swabs (Copan, Brescia, Italy) were used to swab surfaces of about 10 × 10 cm^2^. After sampling, swabs were placed in sterile tubes with transport medium, kept on ice during transport to the laboratory, and analyzed within 24 h from collection. The presumptive presence of *B. cereus* was assessed by striking the swabs onto PEMBA (Scharlab, Barcelona, Spain), followed by incubation at 37 °C for 48 h. From each positive sample, a presumptive *B. cereus* colony was sub-cultured onto tryptic soy agar (TSA, Scharlab, Barcelona, Spain) at 37 °C for 24 h. Samples of cheese curd and PDO Taleggio were also submitted to the enumeration of *B. cereus*. Briefly, 10 g of sample was ten-fold diluted in chilled sterile diluent solution (0.85% NaCl and 0.1% peptone) and homogenized for 60 s in a Stomacher 400 (Seward Medical, London, UK). Then, appropriate ten-fold dilutions of the homogenates were made in chilled saline, and spread onto PEMBA plates. Plates were incubated at 30 °C for 48 h (limit of quantification equal to 10^2^ CFU/g).

### 2.3. MALDI-TOF MS Identification

MALDI-TOF MS identification was performed on all isolates as previously reported [13]. Briefly, well-isolated colonies were spotted on the MALDI plates and covered with 1 µL of ethanol, 1 µL of formic acid, 1 µL of acetonitrile, and 1 µL of saturated α-cyano-4-hydroxycinnamic acid (HCCA) matrix solution. Plates were air-dried and placed in the instrument according to the manufacturer’s instructions. The mass spectra were automatically acquired within 10 min, in the positive linear mode at a laser frequency of 60 Hz with an acquisition range from 1.960 to 20.000 Da. Spectra were imported into the integrated MALDI Biotyper software (version 3.1, Bruker, Billerica, MA, USA) and analyzed by standard pattern matching with a default setting. A score ≥ 2.00 indicated identification at the species level, a score ranging from 1.99 to 1.70 indicated identification at the genus level, whereas any score < 1.70 meant no significant similarity of the obtained spectrum with any database entry. Each isolate was tested in triplicate.

### 2.4. RAPD-PCR

Genomic DNA was extracted from *B. cereus* isolates as previously described [14]. RAPD-PCR fingerprinting of bacterial genomes was performed with the primers RPO2 (5′-GCGATCCCCA-3′), M13 (5-GAGGGTGGCGGCTCT-3), HLWL85 (5′-ACAACTGCTC-3′), and OPE03 (5′-CCAGATGCAC-3′) [13,15]. RAPD-PCR reactions were carried out in 50-µL mixtures containing 1 µM primer, 10 µL of Wonder Taq Reaction Buffer, 2.5 U of Wonder Taq (Euroclone, Milan, Italy), 50 ng of genomic DNA, and sterile ultrapure water up to 50 µL. PCR conditions were set as follows: 30 cycles consisting of 94 °C for 1 min, 36 °C for 1 min, and 72 °C for 2 min, followed by one cycle consisting of 72 °C for 10 min. Genomic DNA extracted from *B. cereus* ATCC 14579 and *B. cereus* ATCC 10987 were used as positive controls for each reaction. The reproducibility of RAPD-PCR profiles was assessed in at least three separate experiments.

### 2.5. Detection of Hemolysins, Hemolysin BL, Diarrheal Toxin, Phosphatidylcholine-Specific Phospholipase C, and Proteases

The ability to produce and secrete hemolysins and hemolysin BL (HBL) was assessed by streaking bacterial cells on blood agar (Columbia agar containing 5% horse-blood, Oxoid, Basingstoke, UK) and sheep blood agar (Columbia agar containing 5% sheep-blood, Oxoid), respectively. Plates were incubated at 30 °C for 18 h. Hemolysin production was checked by visually evaluating the presence of a halo of incomplete hemolysis immediately around the colonies. HBL secretion was tested by observing the formation of an unusual discontinuous zone of hemolysis surrounding colonies [10]. Diarrheal toxin was detected in filtered culture supernatants by using the *B. cereus* enterotoxin-reversed passive latex agglutination (BCET-RPLA, Oxoid, Basingstoke, UK) kit accordingly to the manufacturers. The production of phosphatidylcholine-specific phospholipase C (PC-PLC) was evaluated by agar-diffusion assays by using 0.15% l-α-phosphatidylcholine (Sigma-Aldrich, Milan, Italy) [15]. Protease secretion was checked by seeding bacterial cells on 1.5% skim milk (Oxoid, Basingstoke, UK), followed by incubation at 37 °C for 18 h [16]. The presence of a clear degradation halo around colonies was indicative of the presence of proteolytic activities. Experiments were repeated three times in separate days.

### 2.6. Detection of Toxin-Encoding Genes

For the detection of *B*. *cereus* toxin-encoding genes (sphingomyelinase (Smase), *sph*; diarrheal toxin Bcet, *bcet*; enterotoxin FM, *entFM*; enterotoxin S, *entS*; phosphatidylinositol-specific phospholipase C (PI-PLC), *plcA*; cytotoxin K (CytK), *cytK*; non-hemolytic enterotoxin complex (NHE), *nheA*, *nheB*, and *nheC*), PCR reactions were performed on bacterial genomic DNA. For each gene, amplification conditions were set as previously described [15]. Genomic DNA extracted from *B. cereus* ATCC 14579 was used as positive control for each amplification.

### 2.7. Biofilm Formation

Bacteria were tested for biofilm formation in Luria Bertani (LB) broth (Oxoid, Basingstoke, UK) as previously described [16]. Briefly, bacteria were grown to the early stationary phase in LB at 37 °C and diluted to an optical density at 600 nm (OD_600_) of 0.01 in fresh LB. 2 mL were transferred to wells of polystyrene 24-well plates (Falcon/Becton Dickinson, Franklin Lakes, NJ, USA) and incubated in static conditions for 48 h at 37 °C. At the end of incubation, non-adherent planktonic bacteria were removed, wells washed three times with phosphate buffer saline (PBS), and air-dried. Microbial biofilms were stained with 2 mL of 0.3% crystal violet for 10 min, washed with distilled water, and air-dried. Crystal violet was solubilized with 2 mL of 70% ethanol and the OD_590_ measured. The strong biofilm producer *B. cereus* ATCC 10987 was used as positive control, while sterile LB was used as negative control. Experiments were repeated three times in separate days and two technical replicates were carried out for each assay.

### 2.8. Statistical Analysis

Quantitative data were expressed as mean ± standard deviation (S.D). Statistical analysis was performed on GraphPad Prism version 8.0.2 (GraphPad Software, San Diego, CA, USA). Data concerning the prevalence of *B. cereus* were subjected to chi-square test, in order to compare the results obtained from the different areas of the plant. A comparison was also made among FCS, tr-NFCS, and non-tr-NFCS samples, by applying the exact Fisher’s test. For the evaluation of biofilm formation, the one-way analysis of variance (ANOVA) with Dunnett’s multiple comparisons test was applied, by setting the sterile LB values as control group. A two-sided *p*-value < 0.05 was considered significant.

## 3. Results

### 3.1. Prevalence of B. cereus in Environmental Samples, Curd, and Final Product

**Figure 1:**
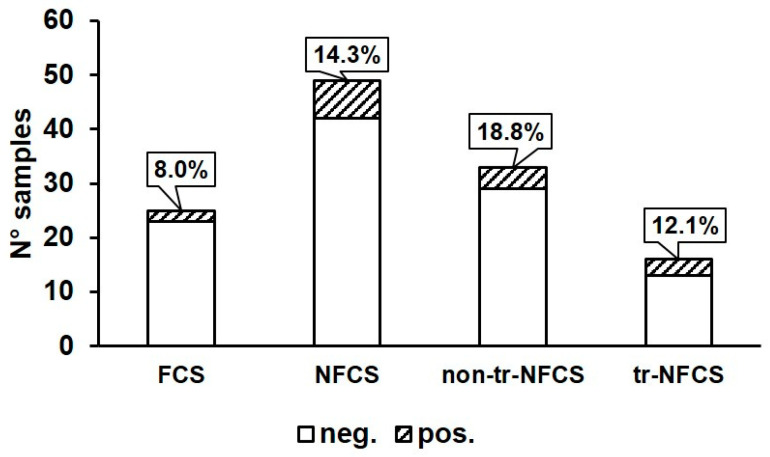
*B. cereus* detection rate (in percentage) in samples obtained from food contact surfaces (FCS) and non-food contact surfaces (NFCS). NFCS samples were also classified as total NFCS (NFCS), transfer-non food contact surfaces (tr-NFCS), and non-transfer-non food contact surfaces (non-tr-NFCS). Neg: samples negative for the presence of *B. cereus*; Pos: samples positive for the presence of *B. cereus*.

In this study, the presence of *B. cereus* was investigated in various environmental sites in a dairy processing plant producing PDO Taleggio cheese during two sampling sessions (May and July). As reported in Table 1, *B. cereus* was isolated from 9/74 environmental sites. A higher prevalence (8/40 samples) was observed during the first sampling session if compared to the second (1/34), but no particular correlation could be inferred because of the low number of positive samples. This difference could be linked to several uncontrollable factors, such as the different application of good hygienic practices in July compared to May. Data obtained from environmental samples showed a similar distribution of *B. cereus* in the different areas dedicated to PDO Taleggio production (4/37 for production area, 2/28 for brining/ripening area, 2/4 for packaging area, *p* > 0.05; Table 1). Sampling points from different environmental sites were classified as food contact surfaces (FCS, N° = 25), transfer-non food contact surfaces (tr-NFCS, N° = 16), and non-transfer-NFCS (non-tr-NFCS, N° = 33). As shown in Figure 1 and Table 1, the positivity rate for *B. cereus* detected on FCS was moderate (2/25) and not significantly different from that found on total NFCS (7/49). Considering the results obtained from NFCS (Table 1; Figure 1), the presence of *B. cereus* was revealed on four non-tr-NFCS (water hose and floor of the production area, and a drain of the packaging area; 4/33) and three tr-NFCS (trolley wheels of the packaging area, operators’ boots of the brining/ripening, and sampler’s overshoes; 3/16), all surfaces related to the floor. Among the FCS, the presence of *B. cereus* was only detected on a vat used for brining and on a duct for the transport of whey.

In addition to environmental samples, the presence of *B. cereus* was also investigated in salt/saline, curd, and in the final product PDO Taleggio (Table 1). *B. cereus* was detected in the cheese curd in both sampling sessions, with amounts equal to 1.6 × 10^3^ CFU/g and 2 × 10^2^ CFU/g in samples from the first and second session, respectively. In contrast, the microorganism was not isolated from salt/saline and PDO Taleggio cheese.

### 3.2. B. cereus Identification and Clustering

**Figure 2:**
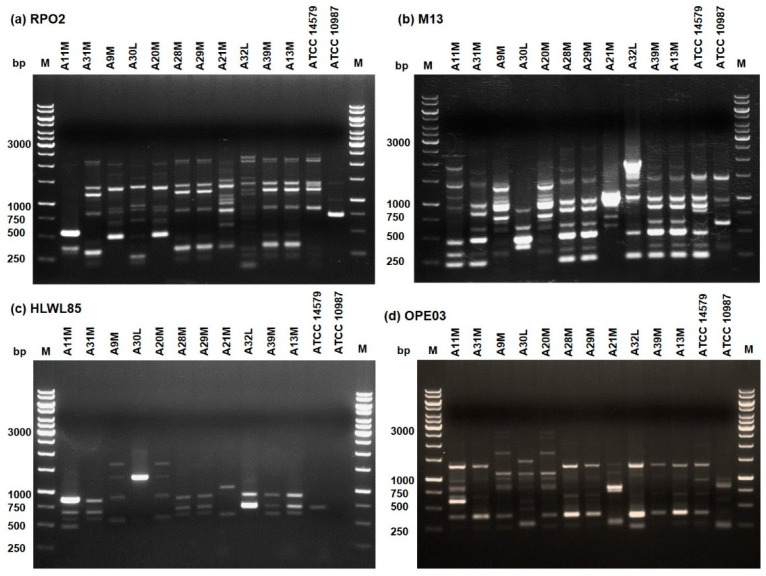
RAPD-PCR amplification profiles obtained with primers RPO2 (**a**), M13 (**b**), HLWL85 (**c**), and OPE03 (**d**) from *B*. *cereus* isolates. Genomic DNA extracted from the reference strains *B. cereus* ATCC 14579 and *B. cereus* ATCC 10987 was used as control of each reaction. M = Thermo Scientific GeneRuler 1 kb DNA Ladder (Thermo Fisher Scientific, MA, USA). Numbers on the left margins of each panel indicate the position of the molecular weight standards (bp).

A total of 11 presumptive *B. cereus* isolates (Table 1) were subjected to identification by MALDI-TOF MS. This technique had been successfully applied for the identification of *B. cereus* obtained from clinical, food, and environmental samples, as well as from probiotic formulations [13,16,17,18]. All isolates were confirmed as belonging to the *B. cereus sensu strictu* species, with scores greater than 2.00. Since the isolates were obtained from different samples of the dairy processing plant, we wondered whether they could represent different *B. cereus* strains. Molecular typing was performed by RAPD-PCR, a method successfully used to genetically differentiate strains belonging to the *B. cereus* species [15]. Primers RPO2, M13, HLWL85, and OPE03 were separately used to amplify the genomic DNA of each isolates and the amplification profiles obtained with each primer were compared (Figure 2). Genomic DNA of *B. cereus* ATCC 14579 and *B. cereus* ATCC 10987 were used as controls for each reaction. Global analysis of the generated patterns (Figure 2) lead us to group the isolates into six different clusters of similarity. Within each group, it can be reasonably assumed that the isolates are a single bacterial strain. The amplification profiles obtained for A11M, A30L, A21M, and A32L were unique (Figure 2), thus indicating that these isolates belong to four different clusters that were called cluster 1, 4, 5, and 6, respectively. Genetic similarities were found for strains (i) A31M, A28M, A29M, A39M, and A13M; and (ii) A9M, and A20M. These results lead us to group the isolates into two different clusters of similarity, named cluster 2 and 3, respectively (Figure 2).

### 3.3. Virulence Potential of B. cereus Isolates

The ability of *B. cereus* strains to produce/secrete virulence factors or enzymes with a potential spoilage activity (PC-PLC, proteases, hemolysins, and HBL) was investigated by visualizing the activity of these virulence factors on solid media containing skim milk, phosphatidylcholine, horse blood, and sheep blood, respectively. BCET-RPLA kit was used to detect the presence of the diarrheal toxin (i.e., the L_2_ component of HBL [19]) in culture supernatants. As shown in Table 2, all *B. cereus* strains produced PC-PLC, proteases, and hemolysins, while HBL activity, as well as the L_2_ component of HBL, were detected in clusters 1, 2, 4, and 6. Cluster 3 was unable to produce L_2_ and this result correlated with the absence of the typical discontinuous halo of HBL on sheep blood agar found for this group. Interestingly, while L_2_ was detected in culture supernatants, no HBL activity was observed for cluster 5 (Table 2), suggesting that this group could not produce at least one of the other HBL components.

When specific tests were not available, we used PCR to search the toxin-encoding genes (*nheA*, *nheB*, *nheC, sph*, *plcA*, *cytK*, *bcet, entFM*, and *entS*, encoding NHE_A_, NHE_B_, and NHE_C_, Smase, PI-PLC, CytK, diarrheal toxin Bcet, enterotoxin FM, and enterotoxin S, respectively) in *B. cereus* genomes. The analysis revealed that all clusters possessed in their genome the *nheA, plcA*, *cytK*, and *entFM* genes (Table 2). On the other hand, clusters 1, 2, 3, and 6 carried also *nheB* and *nheC*, while cluster 4 had *nheB* but not *nheC*. Interestingly, cluster 5 did not possess *nheB* nor *nheC*. Since NHE requires all the three components to be active, these results indicate that clusters 4 and 5 do not produce NHE. Most clusters carried *sph*, with the only exception of group 5. The *bcet* and *entS* genes were present in the genome of strains belonging to the clusters 2, 4, and 6, while were absent in those of the clusters 3 and 5. Cluster 1 carried *entS*, but not *bcet*.

**Figure 3:**
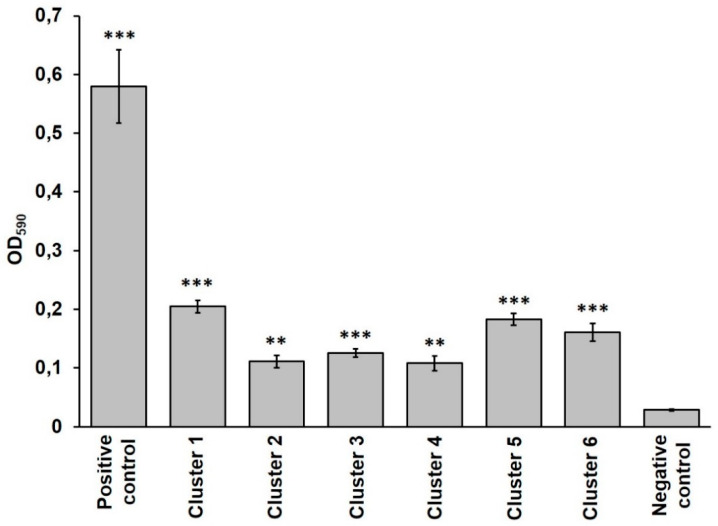
Biofilm formation by each *B. cereus* cluster evaluated by the crystal violet assay (OD_590_). Positive control: *B. cereus* ATCC 10987; negative control: sterile LB. Data are expressed as mean ± S.D. ** *p* < 0.01, and *** *p* < 0.001 compared to the negative control.4 Discussion.

The ability of the six *B. cereus* clusters to produce microbial biofilms was evaluated by the crystal violet assay. This analysis indicated that all strains were able to form biofilm (*p* < 0.01 for cluster 2 and 4 and *p* < 0.001 for cluster 1, 3, 5, and 6 compared to the negative control; Figure 3), although its amount was lower (*p* < 0.001) compared to the strong biofilm producer *B. cereus* ATCC 10987 (positive control, Figure 3).

*B. cereus* pathogenic potential is complex and is primarily due to the secretion of several toxins and tissue-destructing exoenzymes [10,11,12]. These virulence factors include hemolysins, cytotoxin (i.e., CytK), trimeric complexes (i.e., NHE and HBL), proteases, enterotoxins (i.e., enterotoxin FM, diarrheal toxin Bcet, and enterotoxin S), and phospholipases (i.e., PC-PLC, PI-PLC, and Smase). The presence of potentially virulent *B. cereus* strains in dairy processing plants represents a serious health risk for the consumers, because of their ability to cause foodborne diseases. In addition, the production of lipases and proteases by *B. cereus* could lead to food degradation and spoilage, thus resulting in a great economic loss for food producers [20].

This study analyzed the degree of diffusion of *B. cereus* in the Taleggio production environment, considering a plant typology where all the phases (production and ripening) are performed following the PDO standard. The microorganism was found in all the areas of the plant at similar rates. This result was expected for the ubiquity of the microorganism and the multiplicity of contamination sources [21]. As regards the microbe distribution in different areas, its recovery from transfer-non food contact surfaces (tr-NFCS) suggests that these surfaces represent contamination carriers between different working areas or from the outside (e.g., during the working pauses or trucks loading/unloading). Water hoses (considered as non-tr-NFCS), which are frequently handled by the operators, can represent both an end point and a potential source of contamination. The presence of *B. cereus* on the whey duct was considered very critical, since whey is frequently used for products in which the presence of this potential pathogen could be dangerous, such as ricotta or products intended for infants [6,22]. No contamination of the curdling tank was evidenced, suggesting the absence of *B. cereus* spores in the pasteurized milk used for production. In fact, the total absence of spores cannot be assured by the producer and requires proper control procedures during cheese manufacturing [22,23,24].

The isolation of *B. cereus* from curd but not from Taleggio was considered interesting. In fact, curd can represent a suitable substrate for *B. cereus* to replicate and potentially reach counts of concern. It is arguable that the early addition of pro-technological starter cultures (*Lactobacillus bulgaricus* and *Streptococcus thermophilus*) coupled with curd and cheese salting contrast the growth of microbial contaminants during the first drying and ripening phases of Taleggio cheese, as observed for other dairy products [5,25,26,27,28]. In this light, *B. cereus* contamination of the brining vat could be considered as non-critical. In fact, differently from other bacterial species [29,30], *B. cereus* shows difficulties in replication on the PDO Taleggio cheese surface with the increasing ripening period [7].

The presence of different clusters of *B. cereus* strains in the same plant suggests the possibility of a reiterated entry of the microorganism from external sources. It is interesting to highlight that cluster 2 comprises isolates found on the floor of the production area, on operators’ boots in the ripening area, on trolley wheels in the packaging area, on the overshoes of a person that was performing the environmental samplings, and in the cheese curd (Table 1). Thus, this strain shows an evident circulation and easy spread from a section to another. A similar correlation was found also for the isolates belonging to cluster 3, since they were found in two different places (the internal surface of whey drainage ducts and water hose, respectively) of the production area (Table 1).

The isolates obtained from the analyzed Taleggio production plant showed a constant ability to produce PC-PLC, proteases, and hemolysins, whereas only most of them were able produce HBL (66.7%). These results agree with previous studies showing a very high rate of PC-PLC, protease, and hemolysin activity [4,31,32,33], and a variable rate of HBL production (ranging from 20 to 90%) by *B. cereus* isolates [21,34,35].

Considering cheese, several studies confirm the high prevalence of *nhe* genes in *B. cereus* isolates [21,31,32,35,36,37,38]. In this study, we found that all the strain clusters harbored *nheA*, and some also *nheB* (83.3%) and *nheC* (66.7%), indicating that 66.7% of the strains are potentially able to secrete active NHE. The frequency of *cytK* observed in this study (100%) was markedly higher than that observed in cheese by other authors [37,38]. A high frequency of CytK production is generally reported for food poisoning or food related strains [33,39]. The additional finding that all the strain clusters harbored the *entFM* gene and some also *bcet* (50.0%), and *entS* (66.7%) agree with those reported by previous studies performed on milk and dairy products [31,33,37,38].

Together with spores, biofilm production is a pivotal factor for *B. cereus* survival and persistence in the food industry and dairy processing plants, acting as a barrier against the action of chemicals and sanitization procedures [40,41]. The formation of these microbial consortia favors *B. cereus* adhesion to several FCS and NFCS surfaces, thus resulting in a potential source of cross-and post-processing contamination of finished products [42]. All the *B. cereus* strains isolated in this study were able to form biofilm. This evidence supports the idea that spreading of certain *B. cereus* strains among different sites of the plant could be, at least in part, promoted by their ability to form biofilm. However, the reduction in the number of *B. cereus* strains found in the second sampling session compared to the first was strongly indicative that cleaning and sanitation procedures were correctly applied to remove the potential source of contamination.

## 4. Conclusions

The results of this study highlight the diffusion of potential pathogenic *B. cereus* through a PDO Taleggio processing plant. The ubiquity of this microorganism in the environment was evidenced by the detection of the same strain in different points of the facility, favored by the contamination of equipment that can reach different areas of the production plant, as well as the detection of different strains in the same plant. The presence of *B. cereus* in the curd, coming from multiple environmental sources or from milk, appears not to be completely avoidable. Nevertheless, the absence of the microorganism in the final product, coupled with the unsuitability of the ripened product for *B. cereus* growth, should be regarded as positive factors, suggesting that proper management of the production and initial ripening phases by the producer can assure a high level of consumer’s protection.

## Figures and Tables

**Table 1:** Detection of *B. cereus* in different areas and sample typologies of the dairy plant. FCS: food contact surfaces; tr-NFCS: transfer-non food contact surfaces; non-tr-NFCS: non-transfer-non food contact surfaces.

Area	Typology	Sample	N° Positive/Analyzed Samples	Identified Isolates (Cluster Number *)
**Production**	FCS	Curdling tank	0/3	−
		Table-grind-box-whey duct (internal surface)	1/7	A9M *(3)*
		Operator’s hand	0/2	−
	tr-NFCS	Door-handle	0/1	−
		Trolley wheels	0/3	−
		Operator’s boots	0/2	−
	non-tr-NFCS	Table-boxes- whey duct (external surface)	0/5	−
		Water hose	1/2	A20M *(3)*
		Wall (drip)	0/2	−
		Floor	2/4	A11M *(1)*, A13M *(2)*
		Drains	0/6	−
	**Total**		4/37 ^#^	−
**Brining/Ripening**	FCS	Brining vat	1/2	A21M *(5)*
		Brushing table	0/2	−
		Brush	0/2	−
		Operator’s gloves	0/2	−
		Boxes-board-cheese cloth	0/5	−
	tr-NFCS	Operator’s boots	1/2	A28M *(2)*
		Plastic doors	0/1	−
	non-Tr-NFCS	Water hose	0/1	−
		Floor	0/6	−
		Drains	0/5	−
	**Total**		2/28 ^#^	−
**Packaging**	tr-NFCS	Trolley wheels	1/2	A39M *(2)*
	non-Tr-NFCS	Drain	1/2	A30L *(4)*
	**Total**		2/4 ^#^	−
**Sampler overshoes**	tr-NFCS		1/5	A31M *(2)*
**Salt/ Saline**			0/3	−
**Curd**			2/2	A29M *(2)*, A32L *(6)*
**Final product (PDO Taleggio)**			0/4	−

* strains were clustered by RAPD-PCR (*c.f.r.* 2.4). ^#^ Data obtained from different areas of the plant were subjected to the chi-square test. No significant differences were found (*p* > 0.05).

**Table 2:** Production of PC-PLC, proteases, hemolysins, L_2_ component of HBL, and HBL by each clusters evaluated by phenotypic tests and detection of toxin-encoding genes in bacterial genome performed by PCR.

	Cluster 1	Cluster 2	Cluster 3	Cluster 4	Cluster 5	Cluster 6
**Production of virulence factors evaluated by phenotypic tests**						
**PC-PLC**	+	+	+	+	+	+
**Proteases**	+	+	+	+	+	+
**Hemolysins**	+	+	+	+	+	+
**L_2_ component of HBL**	+	+	−	+	+	+
**HBL**	+	+	−	+	-	+
**Detection of toxin-encoding genes performed by PCR**						
***nheA***	+	+	+	+	+	+
***nheB***	+	+	+	+	−	+
***nheC***	+	+	+	−	−	+
***sph***	+	+	+	+	+	+
***plcA***	+	+	+	+	+	+
***cytK***	+	+	+	+	+	+
***bcet***	−	+	−	+	-	+
***entFM***	+	+	+	+	+	+
***entS***	+	+	−	+	−	+
